# Lived experience of infertility among Hong Kong Chinese women

**DOI:** 10.1080/17482631.2018.1554023

**Published:** 2018-12-25

**Authors:** Mimi MH Tiu, Juliana YF Hong, Vincent S Cheng, Connie YC Kam, Bernadette TY Ng

**Affiliations:** a School of Nursing and Health Studies, Open University of Hong Kong, Kowloon, Hong Kong; b School of Arts and Social Sciences, Open University of Hong Kong, Kowloon, Hong Kong

**Keywords:** Infertility, lived experience, phenomenological study, Chinese women

## Abstract

**Purpose:** This study aims to explore and describe the phenomenon of women with infertility and to enhance understanding on how infertility affects their lives and the specific social consequence they encountered. **Method:** A qualitative phenomenological design was adopted in this study. A total of 13 women who are infertile participated in the study. A snowball sampling method was adopted. Data were analysed through thematic analysis. **Results:** Four themes emerged in the study, including (i) non-escapable cultural burden in Chinese family; (ii) psychological distress: isolation caused by envy; (iii) disappointment towards reproductive health services; and (iv) self-compassion and religion as coping strategies. **Conclusions:** The causes of infertility are highly complex. Apart from medical conditions, many social conditions would also probably trigger the difficulty of conceiving. Health care professional should also focus on the social and psychological aspects of women of infertility.

## Introduction

Infertility is a major life problem for many couples and probably one of the unfavourable issues affecting one in six couples in Hong Kong. Health care professionals generally consider infertility as a medical problem with psychological consequences rather than a sociocultural constructed issue. However, the emotional effect of infertility creating problems for infertile couples and tormenting the affected family and society cannot be ruled out (Hasanpoor-Azghdy, Simbar, & Vedadhir, 2014; Jafarzadeh-Kenarsari, Ghahiri, Habibi, & Zargham-Boroujeni, 2015).

Women seem to be mainly impacted by infertility. Previous researchers have suggested that women are actually more adversely affected by infertility than men (Ryan, 2009; Wu & Hart, 2002). Women are commonly marginalized, stigmatized and socially excluded (Gillespie, 2000; Letherby, 2000; Park, 2002). Particularly in the Chinese community, women are frequently blamed for their inability to conceive. The pain and loss associated with infertility notably lead to psychological consequences, which can influence every aspect of an infertile woman’s life, including her behaviour, marriage and social relations, economic and cultural consequences (Ismail & Moussa, 2017; Lindsey & Driskill, 2013). Naturally, women strongly refer to infertility as a life crisis and the most stressful experience in their lives (Cheng, Stevenson, Yang, & Liou, 2018; Freeman, Boxer, Rickels, Tureck, & Mastroianni, 1985; Ismail & Moussa, 2017).

Therefore, infertility is an unanticipated life-course disruption to women (Ulrich & Weatherall, 2000) as it can lead to tremendous societal repercussions other than personal suffering. Social effects related to infertility can profoundly affect a woman’s emotional well-being and life chances (Goffman, 1968). Questions such as “How are infertile women seen by others?” and “How do they see themselves?” are actually socially constructed, which will lead them ultimately to conclude that they are standing at the edge and unable to achieve a desired social role.

Thus, an important issue should attract full and scrupulous attention from society. To our knowledge, studies have neither captured the experiences of infertile women in the sociocultural context nor investigated the social consequences and resulting life of infertile women in Hong Kong with Chinese citizens as the majority.

Moreover, a continuous decline in birth rate impedes population increase as it tightens the workforce, which in turn hampers the long-term and overall productive capacity of a society. When observing the infertility trend in Hong Kong, which has remained low in the past decades, Hong Kong now faces a crucial situation. The low birth rate of the city will not only affect its economic growth, but also accelerate its ageing population. Under a nosediving birth rate, women suspected of infertility should receive reproductive treatment as early as possible to improve their chances of childbearing. Moreover, the government should provide a supportive environment to boost women’s fertility. However, despite the high demand for infertility services in Hong Kong, infertility treatment is only provided at 9 public hospitals to women up to the age of 40. Furthermore, the waiting time of up to three years is unbelievably long.

## Research methods

### Design

This study adopted a qualitative phenomenological study to discover, explore and describe the phenomenon of women with infertility. This research is also an attempt to gain a better understanding of the processes in which infertility affected their lives and the kinds of social consequences they experienced. Phenomenology is the most appropriate methodological approach for this study as it describes experience as lived by people. The end result of this study is a thick description regarding the lived experience related to the phenomenon of infertility.

### Sampling method and selection criteria

Infertility is a disease of the reproductive system defined by the failure to achieve a clinical pregnancy after 12 months or more of regular unprotected sexual intercourse. (Gurunath, Pandian, Anderson, & Bhattacharya, 2011; WHO-ICMART glossary, 2009). According to the International Committee for Monitoring Assisted Reproductive Technology of the World Health Organization, there are two types of infertility (Zegers-Hochschild et al., 2009):

1. Primary infertility refers to couples who have not become pregnant after at least one year having sex without using birth control methods.
2. Secondary infertility refers to couples who have been able to get pregnant at least once, but now are unable.

Hong Kong Chinese women who still have a menstrual cycle and fail to achieve a clinical pregnancy after 12 months or regular unprotected sexual intercourse were recruited to participate. Women suffering from both primary and secondary infertility were included in this study.

Snowball sampling was employed as this approach effectively locates information-rich key informants. This method is most frequently used when the population comprises people with characteristics that are probably difficult to identify, such as being infertile. Therefore, apart from the first informant who is a friend of one of the researchers, other potential people with a similar trait of interest are identified and referred to participate in the study by the assistance of the informants.

### Data collection

In-depth interviews are optimal for collecting data on individuals’ personal perspectives and experiences, particularly when sensitive topics are explored. The interviewer conducted a one-to-one in-depth semi-structured interview, and an open-ended style was employed to encourage informants’ free expression of their personal experiences. This study was conducted in Hong Kong from November 2016 to October 2017. The data collection process was as follows.

Before commencing the interview, the interviewer approached the potential informants through telephone contact and performed a preliminary assessment of the informants’ medical and fertility history by asking them questions such as age, occupation, education level and years of marriage, as well as history of prior pelvic inflammatory disease, uterine and tubal abnormalities and family history of infertility. During the interview, the interviewer, according to the interview guide, raised open-ended questions and took notes together with tape-recording. Each interview, lasting for about an hour, was closed when the data reached a saturation point. After the interviews, the interviewer had to follow up the informants in case of missing data or clarification of ambiguous data. Since the interviewer is an experienced nurse, emotional upset from informants could be detected immediately and appropriate counselling could be provided without delay.

A total of 15 potential informants were recruited. However, two women who were listed as potential informants before finally refused to participate in the study as they considered the study was too personal and did not intend to disclose sensitive personal matters. At the end, 13 Hong Kong Chinese female informants aged from 34 to 52 and married for 4 to 22 years were interviewed (see Table 1).

**Table 1.** Demographic characteristics of informants.

Informant Number (Code)	Age	Academic level	Employment	Year of Marriage	Religion	Type of Infertility
1 (Inf-1)	39	Bachelor degree	No	9	Yes	Primary
2 (Inf-2)	50	Bachelor degree	Yes	12	Yes	Primary
3 (Inf-3)	42	Doctoral degree	Yes	16	Yes	Primary
4 (Inf-4)	44	Bachelor degree	Yes	16	No	Secondary (miscarriage)
5 (Inf-5)	34	Bachelor degree	Yes	5	No	Primary
6 (Inf-6)	50	Bachelor degree	Yes	20	Yes	Secondary (miscarriage)
7 (Inf-7)	44	Bachelor degree	Yes	12	Yes	Primary
8 (Inf-8)	38	Bachelor degree	Yes	9	Yes	Primary
9 (Inf-9)	37	Bachelor degree	Yes	9	Yes	Secondary (miscarriage)
10 (Inf-10)	34	Bachelor degree	Yes	4	Yes	Primary
11 (Inf-11)	42	Master degree	No	8	No	Secondary
12 (Inf-12)	52	Bachelor degree	No	22	Yes	Primary
13 (Inf-13)	39	Bachelor degree	Yes	7	Yes	Primary

### Setting of data collection

Interviews were conducted in person. The time and venue for the interview, held in the informant’s residence, the university’s meeting room or a quiet corner of a café, were entirely proposed by the participants according to their convenience.

### Semi-structured interview

An interview guide was developed to cover the physical, psychological and social implications of infertility. The semi-structured questions for interview were:

1. Could you tell me how you came to be diagnosed with infertility?
2. Does the diagnosis reveal which side causes infertility, your side or your husband’s?
3. How do you feel after realizing that you have a fertility problem?
4. Have you sought assisted reproductive technologies? If yes, describe your feeling throughout the entire process.
5. Have you experienced any pressure caused by childlessness and how have you handled it?
6. How do you see a family structure?
7. What kind of support would you like to receive?

### The interviewer

A part-time registered nurse, holding a masters degree in health promotion and having extensive experience in interviewing was recruited for this research project. She was responsible for a series of interview-related duties including the approach of potential informants, scheduling the appointments, explaining to them the details of the study and obtaining their consent, as well as the conduction of a preliminary screening of the informants.

### Pilot study

Findings from a pilot study can guide the design and implementation of an efficacy study (Leon, Davis, & Kraemer, 2011). Through the pilot study, it aimed to examine the appropriateness of the interview guide, to assess the feasibility of the procedures and to identify modifications in the further study. The pilot work in this study was regarded as the preparation of the major study. Through it, modification of the interview guide was made and interview questions were revised and strengthened. More important, it helped the interviewer become more familiar with skills in raising sensitive topics and exploring questions to elicit more in-depth details. One informant was included in the pilot study and the snowball sampling had also been trialled.

### Data analysis

Once the interviews were transcribed verbatim, the researchers read each transcript, made notes in the margins of words and performed an open coding on the statements in the text. Thematic analysis was employed to identify themes and categories that emerged from the interview transcripts. All data were reviewed by the entire research team to ensure the original meaning of the scripts was accurately translated from Chinese to English.

## Results

In this study, four major themes were generated, namely, (i) non-escapable cultural burden of Chinese family; (ii) psychological distress: isolation caused by envy; (iii) disappointment towards the reproductive health services; and (iv) self-compassion and religion as coping strategies.

## Theme 1: Non-escapable cultural burden of Chinese family

The family in Chinese culture was not only a social unit; the purpose of the family is the continuation of the family line. If a woman is unable to bear a child, that devastating problem in somehow can be referred as violation of filial piety. Informants in general still concerned about whether desires of their parents or in-laws would be annoyed and stated that they felt like a failure with their inability to provide their parents and in-law with a grandchild to love. An informant stated: ‘When I told my mother-in-law that I was unable to have a child, she was very disappointed’ (Inf-10).

Moreover, infertile women always felt guilty for being unable in discharging of their obligations to continue the family line: ‘I felt under pressure as my husband’s family is from Chiu Chow. They are very much concerned about having their bloodline to pass down through generations’ (Inf-10). Also, informants considered having children was a way to maintain a harmonic inter-family relationship:

My husband’s grandma is ninety years old, almost every time I met her she keeps asking me if I have got pregnant. She is so desperate for seeing me to bear a child. I really think a child can keep the lineage of a family. (Inf-3)

In contrast, some informants reported that their in-law was outraged about their infertility and turned negative toward them:

My mother-in-law questioned me: You had attained a high education level but only assumed a part-time job soon after marriage. Why didn’t you try every means to bear a child? (Inf-2).

My mother-in-law has two sons. She always makes the comparison between me and the other daughter-in-law. She told me, ‘Ah Ling [another daughter in-law] is such a good daughter-in-law, she gave me 3 lovely grandchildren, you should learn from her … and don’t be workaholics …’. I was so upset. (Inf-8)

## Theme 2: Psychological distress: isolation caused by envy

Similar to previous studies (Graham, Hill, Shelly, & Taket, 2011; Greil, Slauson-Blevins, & McQuillan, 2010; Williams, 1997), psychological distress including loss, anxiety and depression, a feeling of hopelessness and loss of control, anger and resentment and low life satisfaction were noted in the interviews with 13 infertile women in this study. Informants in some ways experienced negative solitude:

I withdrew from all my relationships with those colleagues who have children and kept away from those women who had just given birth to babies (Inf-5). Another informant even indicated that: I would not surf any Facebook postings if my friend has given birth to a baby. I hate seeing those baby photos. (Inf-4)

Informants reflected that they suffered, to a certain extent, from envy and resentment and the bitterness could even extend to good friends and children. When their friends joyfully talk about their children, some informants would have a hard feeling in their heart and they expressed that their internal turmoil of guilt and jealousy could be deepened when getting together with children.

My best friend, whom we knew each other when we were 8 years old, [sic] told me that she was pregnant soon after her marriage. I was not happy or excited about that. I did not even congratulate her. (Inf-7)

I enjoy taking care of my friend’s daughter. However, I find myself getting increasing upset and experience a kind of sadness after playing with the children. I wish I could receive such good luck of having such lovely girls. (Inf-12)

## Theme 3: Disappointment towards reproductive health services

Health professionals, particularly those employed in infertility clinics, should be in the best position to provide supports for patients facing an infertility crisis. In this study, informants frequently complained about health professionals, accusing that they were somewhat dehumanized and demonstrated negative concern over the participants’ feeling of helpless or their struggles with a hard time in life:

The doctor did not support me much; it seemed like a production line; he just kept me informed about the whole process: when to have an injection, when to have the embryo implanted and when to have the pregnancy test again if conception failed. He might get used to handling patients with the same problems all the time that he no longer showed much concern for me and cared about my psychological state at all. (Inf-10)

The fertility services were criticized for not being client-oriented. Even the nurses did not show any compassionate care to the patients throughout the process of treatment:

The health care professionals did not care about your emotion. They just treated every patient as a number and their tone of voice was always indifferent. Even the nurses were cool with poker faces. After a lengthy and afflictive time for the investigation results, the nurse just informed me about a number of data over the phone. (Inf-9)

Infertility treatment in Hong Kong is categorized as a non-core service in the public health sector. However, fertility services in private health sector are costly. Due to financial concern, some infertile women in Hong Kong have no alternative but to receive the relevant services in nearby countries:

I went to Taiwan for the treatment as the cost is less expensive there. However, I felt very lonely as I have to stay in Taiwan for nearly a month and go through the procedure on my own without my husband and family members’ support. (Inf-1)

## Theme 4: Self-compassion and religion as coping strategies

### 4.1 Being self-compassionate

Infertile women have suffered to a certain degree from psychological distress and anguish. Fortunately, they successfully dredged up their emotional dissonance and transferred the crisis to a positive attitude. They tempted to overcome the negative emotions and admit openly the pain. More importantly, they went through their suppressed pain and tried to take up a more positive attitude towards infertility. They would think about the benefits of having a child-free life, more free time, saving more money and enjoying leisure-time interests or hobbies:

The competitive education system of Hong Kong is getting even worse. To let their children enter an ideal school, more and more parents put extra pressure on their children. I feel quite relaxed for the time being as I do not have any child. (Inf-13)

Without kids, I can enjoy my life: I can learn different languages, join singing class and learn how to play different instruments. If I have kids, I guess they will be the ones to learn these. (Inf-3)

### 4.2 Having a religion

Crisis situations such as infertility can disrupt the normal life of a woman. Religion can give an individual the benefit of psychological stability. The majority of the informants in this study believed in religion. They rather firmly believed that their devout prayers, especially their unbreakable faith in God, would ultimately give them strength to overcome the crisis or enable them to conceive:

Everything we encountered has causes and effects, and God arranged all the events for us. I hold this belief. I endured the pressure and pain for six to seven years before accepting the fact that I am infertile. (Inf-8)

I trust that God will give me a baby someday … someday when God thinks I am ready to be a good mother. (Inf-5)

## Discussion

Hong Kong is one of the most densely populated cities in the world with a population as high as 7.3 million, with 92% comprising Chinese citizens (Hong Kong Census & Statistics Department, 2017). A demography such as this in Hong Kong should be rich in Chinese culture and civilization. However, as a British Colony for 156 years, Hong Kong has previously developed its distinctive trait mingling with eastern and western cultural characteristics. One of the noticeable phenomena is the Hong Kong people’s ability to balance their modernized way of life with traditional Chinese practice.

In Chinese culture, the structure of the family has traditionally followed a hierarchical order, where deference and obedience to elders are still of paramount importance; the family is basically dominated by the male members. Moreover, the family name was carried on only through the male members of the family. Therefore, having a son as heir in the Chinese family is somewhat indispensable (Challen, 1967). The belief’s partial rootedness in Confucianism tenets carries a special meaning to the Chinese family. Although the Chinese culture has dramatically changed in recent years, the family structure and the importance of family values remain valid.

From the sociological perspective, incapacity to bear a child is not only a biological issue. Previous studies have identified two main sociocultural factors, namely, pronatalism and patriarchy, affecting one’s experience of infertility. Pronatalism is the ideology which suggests that a person’s social value is linked to procreation (Parry, 2005). Pronatalism can be a strong social force causing social degradation and stigmatization of infertile couples. It is still a distinct social force that influences some Chinese women’s reproductive decision-making (Petropanagos, 2017; Wu & Hart, 2002). An ancient Chinese proverb mentioned that having no descendants is the most unfilial duty of a son. Building a family with children is the norm in traditional Chinese values (Ng, Liu, Chan, & Chan, 2004). Although the younger generations of Chinese are less conservative today, a few of them still desire to have their own children to carry the family name (Tong & Fong, 2014). This study revealed that most of the participants embraced a fusion of Chinese and Western cultures and laid no emphasis on having a child to carry the family name. However, they obviously displayed strong childbearing desire as they explained their intention to fulfil the motherhood role and establish a conjugal family.

The experience of infertility is difficult to express as it interferes with nearly every aspect of the sufferers’ lives and relationships. Frequently, people solely focus on the physical causes of infertility but easily overlook the psychological effects. Infertile women constantly carry physical, social, and emotional burdens (Hasanpoor-Azghdy et al., 2014). Notably, the grief associated with all these burdens is complicated. Among infertile couples, males and females should more or less be equally held accountable for infertility. Unfortunately, the social burden generally falls disproportionately on women, who exclusively bear the blame for their inability to conceive a child and experience the emotional turmoil mixed with anxiety and feelings of guilt, shame, grief and fear (Lindsey & Driskill, 2013; Loke, Yu, & Hayter, 2011). Most infertile women generally feel disappointed, depressed and isolated, especially when friends or relatives gather around to welcome them back to their families after the confirmation of their infertility. Informants in this study shared similar experiences and confided that envy would compel them to intentionally distance themselves from children.

In Hong Kong, services provided for the treatment of infertility are limited and costly. They are far beyond the needs of a large proportion of infertile couples desperately yearning for assistance. Simultaneously, medical insurance is incapable of offering any assistance, despite probably having no relevance to the situation as most insurance policies do not cover this kind of intervention.

Despite the relatively lowered price of the fertility services provided by the Hong Kong government, stringent criteria for selection are set simultaneously for those expressing interest to apply for In Vitro Fertilization (IVF) treatment in public hospitals. Applicants must not exceed the age of 40 at the time of treatment, and each eligible couple must be legally married without any living child from the current marriage. Every qualified female will only be entitled to have three IVF treatment cycles in the public health service (Hospital Authority, 2003). Moreover, as infertility is classified as a low priority service under the Hong Kong public health care system, the waiting list is unacceptably long. The waiting time for the assessment of new cases in the public infertility clinic can take as long as two or even three years. A long waiting time will significantly reduce the treatment success rate with age.

Under public health services, resources and funding for infertility treatments are unacceptably poor as the expensive operation and maintenance costs are only included in the individual Obstetrics and Gynaecology Department budget without additional funding (Cheung, 2015). Considering the insufficient funding, the long-term financial sustainability of the treatments is therefore questioned. Exacerbating the deplorable situation are skilful personnel and experts in infertility treatments leaving the public health service to join the private sector. Apparently, only a limited number of these specialists are available in the market.

Psychological counselling was strongly recommended by the majority of participants, and an infertility self-support group can provide them with a platform to express regret and render emotional, social and practical support for one another (Borman, 1992; Covington & Adamson, 2015). As infertility can be a sensitive topic among family members, any discussion among them might unavoidably disrupt family ties because of different attitudes and perspectives. Patients would rather prefer to share their concerns with others who have encountered the same problems.

Hong Kong has experienced a declining trend in the past 30 years and the total fertility rate of the city is estimated as 1.19. The low fertility rate will be consistently falling well below the replacement level of 2.1 children per woman (Hong Kong Census and Statistics Department, 2015). In view of increasing the birth rate and meeting the high demand of infertility treatment, the Hong Kong government should provide more high-quality fertility care to meet the infertile women’s expectations and needs.

Infertile women in Taiwan commonly used Chinese medicine as a complementary or alternative medicine for treating infertility (Hung et al., 2016). Similar findings were evident in this study as nearly all informants used Chinese medicine in addition to the Western reproductive treatment. Ried and Stuart (2011) in their study suggested that treatment for female infertility with Chinese medicine could improve the pregnancy rates two-fold within 3-6 month period compared with Western medical fertility drug therapy. Chinese medicine for enhancing fertility is non-invasive and safe. It offers a better success rate with relatively lower cost. The government should therefore increase the number of Chinese medicine clinics for infertility.

In the cognitive dissonance theory, Festinger (1957) suggests that, when a state of tension occurs, people will have an inner drive to hold their inconsistent attitudes and beliefs in harmony and avoid dissonance. Motherhood is an expected and valued identity for most women, and failure to bear children is considered deviant in both biological and social aspects and will make the infertile women feel incomplete (Ridgeway & Shelley, 2004; Ulrich & Weatherall, 2000). Self-compassion creates a psychological effect, which can serve as an emotional regulation strategy and a form of resiliency against feelings of self-blame for infertility (Galhardo, Pinto-Gouveia, Cunha, & Matos, 2013; Raque-Bogdan & Hoffman, 2015).

Religious people are often convinced of the power of faith, claiming its ability to empower people to commit actions or achieve goals beyond their limits through their trust in the word of God. Failure in certain infertility treatments would still prompt others to count on God and accept it was a kind of unknown arrangement from their Almighty, notwithstanding that a few might emotionally castigate their God for not responding to their prayers. Seemingly beyond doubt, religion has regularly displayed its capacity to empower people; consequently, health practitioners generally utilize religion in the clinical settings to assist those struggling with a health problem (Oman & Thorensen, 2003).

## Conclusion

The fertility rate in Hong Kong has remained low in the past decades. The adverse effects, such as the ageing population and lack of workforce, gradually emerged in society. Encouragement of fertility can be one of the methods to mitigate the problems, and determining possible assistance to women struggling with infertility is also a positive step to boost fertility.

Stress must be identified. Chronic stress caused by infertility can inhibit the release of sex hormones that would subsequently hamper women’s ovulation and increase the difficulty of conceiving. Therefore, realizing the experiences of those suffering from infertility and scrutinizing the processes in which their experiences have been affected and shaped by the sociocultural context are important.

Prompt and humane treatment must be prescribed. Once the cause of infertility has been diagnosed, the doctor should start infertility treatment as soon as possible because the failure rate will be comparatively higher with age. Consequently, the chance of bearing a child will decline, and the risk of miscarriage will rise. When asking participants related questions on the effect of treatment on their lives, the responses are quite identical. They described that the treatment process was similar to an unfathomable mystery, leaving them clueless about such experience. Apart from those uncomfortable treatment procedures, patients also suffered from physical and psychological pain, such as abdominal pain, bloating, nausea, cervical bleeding, emptiness and loneliness. This finding is correlated to Loke et al.’s (2011) study, which revealed that couples undergoing subfertility treatment reported feelings of incompleteness, guilt, shame and isolation. They were extremely emotional and even broke down without known reasons.

Nevertheless, the causes of infertility are highly complex. Apart from medical conditions, many social conditions would also probably trigger the difficulty of conceiving. To battle a low birth rate and eliminate the stigmatization attached to infertility, focusing only on the support for child-rearing the government and health professionals previously implemented, is inadequate. An overall review of social aspects, such as education, health, living conditions, working patterns and attitude to marriage, is necessary to formulate an appropriate long-term solution.

For the benefit of infertile couples, it is recommended that the health system should review existing policies and determine techniques to improve reproduction services. Thus, an enhanced capability of receiving other infertile women for treatment and the likelihood of success are expected.

## Limitations

A potential limitation of this study is that nearly all the informants had a homogeneous background as in general they came from the middle class, with higher academic achievement, and had religious beliefs. They were able to afford the expensive medical expenses of undergoing medical examination and treatment for infertility. People’s attitude to gender role and relationship varies according to educational level. Moreover, spiritual beliefs play an important role in supporting people when they seek comfort and hope. Future studies should explore the potential relationship between women’s educational level and their meaning of parenthood as well as the effect of copying strategies used by infertile women.
